# A Doctor's Vision

**Published:** 1889-08-24

**Authors:** 


### The Hospital

A Weekly Institutional Journal of

**Science, Medicine, Nursing, and Philanthropy.**

*For Hospitals, Asylums, Medical Practitioners, Students, Nurses, Families, Parishes, Congregations, and the Charitable Public.*

Vol. VI. No. 152.] [August 24, 1889.

### A Doctor's Vision.

What is a vision? A panorama that lies open to the eye of the mind, but which the bodily eye does not yet see. Many men never see visions; do not believe in them; despise those who do. Such men have dense and solid heads, more akin to wood than to human brains. Every man who accomplishes great things sees beforehand. Many medical men in past ages have belonged to the class who never see beforehand. That reproach cannot now be so freely laid to their charge. Mr. Claudius Wheelhouse delivered at Leeds last week his inaugural address as president of the British Medical Association. That address reads like a prophecy: a large, expansive, hopeful prophecy of wholesomeness, safety from dangerous fever, robust health, and long life.

Mr. Wheelhouse sees with his mind's eye a vision which is not the mere creation of an inventive intellect, but a development from assured knowledge, a vision to be realised in actual fact in the experience of succeeding generations. According to this medical prophet, zymotic and exanthematous fevers, those dire scourges of youth and mid-age, are in future to be so entirely under the control of an intelligent sanitary science that they will, at no remote period, be practically extinguished. The speaker could, he said, "foreshadow a time when, by the cultivation of bacteriology and cognate sciences, by a deeper and more profound acquaintance with natural phenomena and laws, the whole range of zymotic and exanthematous diseases would have been subdued and conquered; when the seeds of each would have been isolated, and so studied that their individualities would be recognisable; the soils in which they would grow and those in which they would be sterile would be known and appreciated, and brought under cultivation by the medical men of the day; when the methods of dealing with them would be such that they might be reduced to harmless quantities, and when by the spread of sanitary science the whole human race might be protected from their evil influences. It might not be in our day that these mighty triumphs would be won, but our successors would undoubtedly achieve them; and the time would certainly come when the kingdom of disease would be so closely narrowed down that only the necessary accompaniments of the changes and vicissitudes of natural laws, the evils attendant upon wilful disobedience of those laws, the innumerable accidents to which life and limb must ever be liable, and the inherent defects and deficiencies in the harmonious working of the parts of a machine so exquisitely and delicately constructed as is the frail body which for a time we inhabit, would be the only kingdom in which the professors of medicine and surgery would be called upon to exercise their sway. Then would the victory of their science be complete, and the day would have come when the world would acknowledge that the labours of the physician and the surgeon—patient, enduring, untiring—had reached their final consummation." The vision is hopeful, and the description thereof full of life and spirit. The prophet evidently believes in his own revelation. Has the sober world, which does not possess either medical knowledge or genius enough to see such visions, any ground to hope that this particular one will be realised? That is the practical question for the plain reader. Dreamers of dreams have been innumerable in the world's history. Is this medical dreamer a Joseph whose dreams are sure prophecies of what is to come, or is he a mere babbler who beats the air with vain and foolish talk? Will scarlet fever and diphtheria be banished from the nursery? Will typhoid become a thing unknown except in books? Will typhus and cholera ravage no more, and plagues, with political economy, be relegated to Saturn, or some other more convenient place? The man who, like the president of the British Medical Association, has followed patiently the growth and development of the science of bacteriology, feels and hopes with Mr. Wheelhouse that they assuredly will.

But when and by what means will this thrice happy state of things be brought about? Not in our day, thinks Mr. Wheelhouse. And why not in our day? Because the great world has not enough of sanitary knowledge and sanitary energy to remove and destroy the known causes of bacterial development. That is the whole and sole reason. The means by which so noble a medical prophecy is to be realised in fact are the increase of sanitary knowledge and the strengthening of sanitary energy among the people. A chief part of the mission of The Hospital is to spread that knowledge and to strengthen that energy by all possible means. Would that other newspapers and periodicals which exist for the diffusion of medical knowledge could see it their duty not only to give more light to those who are already within the radius of the sun's beams, but also to extend to the millions who live in sanitary darkness the help of at least a hand-lamp, that they may not all perish together. The idea that the common people cannot understand, and rightly use a knowledge of the common laws of health and disease, is as unphilosophical in itself as it is unworthy of the medical profession of the present time. When doctors had no assured knowledge for themselves, they might be pardoned if they did their best to hide their ignorance from the people. But now, when there is an accumulated body of scientific facts which the most sceptical cannot even question, the exclusive attitude of former and more ignorant ages is both irrational and criminal. Is medicine a science for the people, or is it, like the trade of the shrine-makers of Diana of the Ephesians, a "craft" whereby the initiated "have their wealth"? According as each medical man answers this question for himself, is he worthy of the names of scientist and physician or of charlatan and knave.

The president of the British Medical Association is a man of courage as well as of foresight. Whilst hoping everything for the victory of man over exclusively natural ills, he expressed but little confidence of victory over those ills which result from the indulgence of human passions. There was "one plague-spot," he said, which even the highly-trained practitioner of to-day, and the still more highly-trained practitioner that is to succeed him, would fail to conquer. It would "remain to fester, to kill its thousands, to maim, disfigure, and sap the health of millions, of deserving and of undeserving alike, and, as the great curse of humanity, to baffle all their efforts to arrest the progress of disease, and to render them futile and abortive." His audience "knew the curse to which he alluded, the curse that steadily and unrelentingly pursued the track of licentiousness, of ungoverned passion, of unbridled sensuality. They would admit with him that so long as human nature remained what it was, and was left in unbridled possession of the means of gratification, no ray of light or hope could fall on that dark track . . . the foul stream of syphilis would continue to meander hither and thither and whithersoever it would through the world of life; would poison its springs, would wither even its fairest blossoms, and destroy its richest fruits without selection and without mercy."

There are some who will, perhaps, discount the force of this eloquent and depairing protest, because it is expressed in language which it would hardly seem possible that any facts could justify. The bare truth, however, is that no language can convey to uninstructed readers any adequate knowledge of the wide-spread destructiveness of the disease spoken of by the President of the British Medical Association, or of the suffering and misery which come upon tens of thousands of innocent and guilty alike. Death is a common consequence of this foul disease, both to him who contracts it and to his offspring. Other diseases, worse than even death, may pursue him through many wretched years, whilst deep-seated and all-pervasive constitutional affections may follow his descendants for several generations. Mr. Wheelhouse deserves the gratitude not only of the medical profession, but also of the public, for his grave and serious reference to so widespread a danger. In addressing the British Medical Association he addressed the whole English - speaking world through the medium of the daily press. The long silence which the medical profession has maintained on this subject is now broken, and it may be hoped that many others will be as courageous as Mr. Wheelhouse. To hide such facts as he brought to light in the secret places of professional lore is not only incomparable folly, but the gravest dereliction of public duty. The world cannot live swathed in endless rolls of cotton wool. It is a practical, working, sinning, suffering world, and needs all the light and guidance which every department of science can bring to its aid. Let medicine, as a profession, have done with mysteries and obscurities, and let it take its place side by side with every other organisation which makes for the enlightenment, the happiness, and the practical progress of mankind.

